# Out of Pocket Expenditure for Hospitalization among Below Poverty Line Households in District Solan, Himachal Pradesh, India, 2013

**DOI:** 10.1371/journal.pone.0149824

**Published:** 2016-02-19

**Authors:** Anadi Gupt, Prabhdeep Kaur, P. Kamraj, B. N. Murthy

**Affiliations:** National Health Mission, Government of Himachal Pradesh, Block 6, SDA Complex, Kusumpti, Shimla, Himachal Pradesh, India, 171009; Liverpool School of Tropical Medicine, UNITED KINGDOM

## Abstract

**Introduction:**

Health insurance schemes, like Rashtriya Swasthya Bima Yojana (RSBY), should provide financial protection against catastrophic health costs by reducing out of pocket expenditure (OOPE) for hospitalizations. We estimated and compared the proportion and extent of OOPE among below poverty line (BPL) families beneficiaries and not beneficiaries by RSBY during hospitalizations in district Solan, H.P., India, 2013.

**Methods:**

We conducted a cross sectional survey among hospitalized BPL families in the beneficiaries and non-beneficiaries groups. We compared proportion incurring OOPE and its extent during hospitalization, pre/post-hospitalization periods in different domains.

**Results:**

Overall, proportion of non-beneficiaries who incurred OOPE was higher than the beneficiaries but it was not statistically significant (87.2% vs. 80.9%). The median overall OOPE was $39 (Rs 2567) in the non-beneficiaries group as compared to $ 11 (Rs 713) in the beneficiaries group (p<0.01). Median expenditure on in house and out house drugs and consumables was $ 23 (Rs 1500) in the non beneficiaries group as compared to nil in the beneficiaries group (p<0.01). Non-beneficiary status was significantly associated [OR: 2.4 (1.3–4.3)] with OOPE above median independently and also after adjusting for various covariates.

**Conclusion:**

RSBY has decreased the extent of OOPE among the beneficiaries; however OOPE was incurred mainly due to purchase of drugs from outside the health facility. The treatment seeking behaviour in beneficiaries group has improved among comparatively older group with chronic conditions. RSBY has enabled beneficiaries to get more facilities such as drugs, consumables and diagnostics from the health facility.

## Introduction

Universal health coverage is high on the agenda of many low and middle income countries in the world. One of the major impediments in achieving universal health coverage is the lack of financial coverage leading to high out of pocket expenditure on health care by the households in these countries. [1] Various strategies have been adopted to achieve the universal health coverage such as—reforming tax based health financing or introducing health insurance (HI).[1] HI is one of the methods of providing protection to poor households by reducing out of out of pocket expenditure (OOPE) and improving access to health care. [2]

India spent 4.13% of its gross domestic product (GDP) on healthcare in the year 2008–09.[3] Government’s contribution was only 1.10% of the GDP. Households contributed a significant portion (71.13%) of total health expenditure in India in 2004–05. [3] In India, 30% of the population lived below poverty line (BPL). [4] Hospitalizations have been reported to be the frequent cause of financial catastrophe. Many Indians fell below the poverty line due to hospitalization cost. [5]

Government of India launched two major initiatives to enhance the access to affordable care in the recent years. One was National Rural Health Mission (NRHM) and the other was Rashtriya Swasthya Bima Yojana (RSBY), a health insurance scheme, introduced in 2005 and 2008 respectively. The goals of NRHM, among others, were to improve the public sector health service delivery along with availability of drugs and diagnostics. The objectives of RSBY were to improve access to quality hospital care for BPL households and to protect them financially from catastrophic medical costs. [6] The scheme aimed to improve poor people’s choice of care provider by empanelling both private and public hospitals. Beneficiaries could obtain cashless treatment during hospitalizations as well as in the pre and post hospitalization periods by presenting the smart card at any participating (‘empanelled’) hospital. The scheme was launched in 2008 in Himachal Pradesh, one of the northern states and presently covers all the 12 districts. [6]

The health insurance schemes have three interrelated and separate dimensions: the breadth (number of people covered), the depth (the extent of services covered) and the resulting impacts on health outcomes and financial protection against OOPE. Many studies in different states of India evaluated enrollment of beneficiaries, empanelment of hospitals and the extent of services but the data on reduction of OOPE was limited. In 2013, Solan district in Himachal Pradesh had an enrolment of 75% and had 15 empaneled hospitals. An evaluation of RSBY by the authors in Solan in 2013 suggested a large proportion of beneficiaries incurred OOPE for hospitalizations. However, the proportion and extent of OOPE was not known. So this study was done to document the extent of OOPE among BPL families and to examine whether RSBY reduced the OOPE.

We estimated and compared the proportion and extent of OOPE among BPL families who were beneficiaries and non-beneficiaries of RSBY during hospitalizations in district Solan, H.P., India, 2013. We also determined whether being non-beneficiary was associated with higher OOPE in the study population.

## Methods

### Design and study population

We conducted a cross sectional survey among RSBY beneficiaries and non-beneficiaries. BPL families residing in Solan district, Himachal Pradesh, who had at least one member hospitalized in 2013 were included in the study. Solan district lies in the south of Himachal Pradesh, a northern state of India. The District lies in the foothills of Himalayas having a hilly terrain with an elevation from 300 to 3000 meters above sea level. Solan has a population of about half a million with a population density of 298 inhabitants per square kilometer. More than three fourth of the eligible BPL families are enrolled under RSBY in the district. [6]

We conducted the survey in two groups. The first study group was RSBY beneficiaries residing in district Solan who were members of BPL family and had at least one hospitalization in the facility from January to December 2013 and had been enrolled and beneficiaries under RSBY. The second study group was BPL family members who had at least one hospitalization and did not receive RSBY benefits. In case a respondent was a minor we took either of the parents as respondent. All respondents were part of BPL families’ list, given by the State Government to the insurer for enrollment under RSBY.

### Operational definitions

#### OOPE

Any expense paid by a patient towards healthcare during hospitalization which was not reimbursed under the domains namely drugs, diagnostics, life support system, transport and food.

#### Domains for OOPE

All medications and consumables such as syringes, devices for intravenous infusion, etc. were considered under the drugs. All biochemical, microbiological and pathological investigations were included in the diagnostics. Facilities such as oxygen and blood were defined as life support services.

#### Below poverty line household

A household in the line list of below poverty line families given by the state government to the insurance agency for enrollment for the year 2013.

#### In-house and out house services

Any service provided to the patient from the health facility in which he/she was hospitalized was considered in-house service. The service availed during the course of treatment from outside the health facility was considered out-house services.

#### Acute/chronic conditions

Acute conditions included acute medical (e.g. fever, diarrhea), emergent surgical, injuries and pregnancy related conditions. Chronic conditions included chronic medical and non-emergent surgical conditions.

#### Benefits under RSBY

RSBY is health insurance scheme at the national level managed by Ministry of Labour and Employment, Government of India. The benefits of RSBY extend up to five members of a family which included the head of household, spouse and up to three dependents. Beneficiaries were entitled to hospitalization coverage of up to $ 461(Rupees 30,000) for most of the diseases that required hospitalization. Beneficiaries paid $0.46(Rupees 30) as registration fee while Central and State Governments paid the premium to the insurer, selected by the State Government on the basis of competitive bidding.The scheme required the treatment costs to be reimbursed to the hospital by the insurance company according to fixed rates. All pre-existing health conditions were covered from day one. RSBY reimbursed drugs, diagnostics, consultations fees for doctors a day prior to hospitalization. All drugs, diagnostics, life support services and food were cashless during hospitalization. Drugs for five days from the day of discharge were given free.

### Sample size and sampling procedure

We assumed that 80% population among RSBY enrolled and hospitalized had incurred OOPE, and used absolute precision of 10%, and design effect of 1.5 (actual design effect based on the analysis 1.2) and arrived at a sample size of 91 using Open Epi software. We recruited the same number of respondents in the non-beneficiaries group. We adopted cluster sampling technique and selected 47 of the total 211 Gram Panchayats with the cluster size of two in Solan by using population proportional to size linear systematic sampling. We randomly selected respondents in the beneficiaries group from the line list of RSBY enrolled, hospitalized and benefited individuals from the selected 47 Gram Panchayats. We visited the BPL households in the same cluster till we had identified equal number of respondents in the comparison group who were hospitalized in 2013, but who had not availed the benefit of RSBY, irrespective of enrollment status.

### Data collection

We collected data using a semi-structured questionnaire administered by trained field investigators under the supervision of principal investigator. We collected data on socio-demographic characteristics, enrollment details, treatment details and OOPE for different domains such as drugs, diagnostics, life support services, food and transport.

### Data analysis

We described the characteristics of the households and patients for non-beneficiaries and beneficiaries. We use chi square test to compare the proportions who incurred OOPE overall and in various domains in the non-beneficiary and beneficiary group. We computed the overall median OOPE and domain wise OOPE in three periods. We accounted for any reimbursement while computing the OOPE. We compared the distribution of OOPE (median) in the two groups using non parametric test namely Mann-Whitney U test. We also used t-test to compare the mean age between the groups. We considered p<0.05 as significant for the differences in proportions, means and medians. We estimated the overall median OOPE for the entire study population and defined OOPE above median as the outcome variable. We determined whether non-beneficiary was an independent risk factor for above median OOPE using multiple logistic regression analysis. We used Epi info 3.5.5 for analysis.

### Ethics statement

We obtained approval of Institutional Ethics committee of National Institute of Epidemiology, Chennai and the Government of Himachal Pradesh prior to the study. We provided information sheet to the respondents and obtained written consent from them.

## Results

### General characteristics of the households and respondent

We surveyed 94 respondents each in the beneficiary and non-beneficiary groups. The median household size was five in both the groups. Overall, 24.5% of the household heads were unemployed and 30.9% had never attended school. Nearly 44% lived in kutcha or semi pucca houses and 22.9% did not have access to the toilets. (Table 1) The mean age of the respondent was 46 years in the beneficiaries group compared to 33 years in the other group and was statistically significant (p<0.01). Median number of hospitalizations for the respondent was one in both the groups. Median distance of the hospital from residence was 22 km and 30 km respectively for beneficiaries and non-beneficiaries group. The proportion of male respondents in the non beneficiaries group was 35% and 52% in the other group and was statistically significant (p<0.05). The non-earning respondents were 77(82%) and 59(63%) respectively in the two groups respectively and was statistically significant (p<0.01). Chronic medical conditions were the reason for hospitalization in significantly (p<0.01) higher proportion of beneficiaries (55.3%) as compared to non-beneficiaries (21.3%). Only one respondent was hospitalized for obstetric conditions in the beneficiaries group compared to 37(39%) in the non-beneficiaries group. Proportion of non-beneficiaries who utilized public sector hospitals was higher as compared to non-beneficiaries but it was not statistically significant (90.4% vs. 80.9%, p = 0.096). (Table 2).

**Table 1:** General characteristics of households of non-beneficiaries and beneficiaries, Solan, Himachal Pradesh, India, 2013.

		Non-Beneficiaries (N = 94)		Beneficiaries (N = 94)		Total (N = 188)	
		N	%	N	%	N	%
Occupation of the head of household	Unemployed	30	31.9	16	17.0	46	24.5
	Skilled/Unskilled worker	26	27.7	32	34.0	58	30.9
	Agriculture	33	35.1	35	37.2	68	36.2
	Other Employment	5	5.3	11	11.7	16	8.5
Education of the head of household	Never attended school	34	36.2	24	25.5	58	30.9
	Class 1–5	21	22.3	29	30.9	50	26.6
	Class 6–10	35	37.2	33	35.1	68	36.2
	Higher Secondary & Above	4	4.3	8	8.5	12	6.4
Religion	Hinduism	91	96.8	86	91.5	177	94.1
	Other Religion	3	3.2	8	8.5	11	5.9
Caste	General	45	47.9	44	46.8	89	47.3
	OBC / SC / ST	49	52.1	50	53.2	99	52.7
Type of house	Kutcha	17	18.1	21	22.3	38	20.2
	Semi pucca	24	25.5	21	22.3	45	23.9
	Pucca	53	56.4	52	55.3	105	55.9
Sanitation	Private latrine	72	76.6	73	77.7	145	77.1
	Common latrine / Open defecation	22	23.4	21	22.3	43	22.9

**Table 2:** Socio demographic and treatment characteristics of patients (covariates) of beneficiaries and non-beneficiaries, Solan, Himachal Pradesh, India, 2013.

		Non-Beneficiaries (N = 94)		Beneficiaries (N = 94)		p value
**Socio demographic characteristics**		**N**	**%**	**N**	**%**	
Gender	Male	33	35.1	49	52.1	0.027
	Female	61	64.9	45	47.9	
Education	Never attended school	18	19.1	28	29.8	0.005
	Class 1–5	10	10.6	23	24.5	
	Class 6–10	47	50.0	34	36.2	
	Higher Secondary & Above	19	20.2	9	9.6	
Occupation	Home maker	50	53.2	34	36.2	0.009
	Working	17	18.1	35	37.2	
	Not working	27	28.7	25	26.6	
**Treatment Details**						
Type of treatment	Medical	38	40.4	62	66.0	0.001
	Surgery / Obstetric	56	59.6	32	34.0	
Chronicity of Disease	Acute	74	78.7	42	44.7	0.000
	Chronic	20	21.3	52	55.3	
Type of hospital	Public sector hospital	85	90.4	76	80.9	0.096
	Private hospital	9	9.6	18	19.1	
Knowledge about empanelment of hospital	Yes	44	46.8	80	85.1	0.000
		**Median**	**IQR**	**Median**	**IQR**	
**Number of days of hospitalization (days)**		3	(2–7)	6	(3–8)	0.000
		**Mean**	**SD**	**Mean**	**SD**	
**Age of the patient (years)**		33	16.2	46	17.3	0.000

### Proportions incurring OOPE

Overall, proportion of non-beneficiaries who incurred OOPE was higher than the beneficiaries but it was not statistically significant (87.2% vs. 80.9%, p = 0.319). Proportion of non-beneficiaries who incurred OOPE one day prior to the pre hospitalization was similar in both the groups. Proportion of non-beneficiaries who incurred OOPE for in house and out house drugs and consumables was significantly higher among non-beneficiaries as compared to beneficiaries (73.4% vs. 45.7%, p <0.01) Similar difference was observed for in house and out house drugs for 5 days (72.3% vs. 52.1%, p <0.01). (Table 3) The proportion of respondents who had to borrow money from friends and relatives in the beneficiaries group 59 (67%) was similar to 64 (69%) in the other group.

**Table 3:** Proportion who incurred out of pocket expenditure (OOPE) among non-beneficiaries and beneficiaries, Solan, Himachal Pradesh, India, 2013.

		Non-Beneficiaries (N = 94)		Beneficiaries (N = 94)		
		n	%	n	%	p value
Overall	OOPE (including all domains)	82	87.2	76	80.9	0.319
Pre-hospitalization	Drugs, consumables, diagnostics and consultations one day prior to hospitalization	11	11.7	10	10.6	1.000
During hospitalization	In house drugs/consumables	8	8.5	0	0.0	0.007
	Out house drugs/consumables	62	66.0	43	45.7	0.008
	In house & out house drugs/consumables	69	73.4	43	45.7	0.000
	In house diagnostic	38	40.4	16	17.0	0.001
	Out house diagnostics	28	29.8	34	36.2	0.438
	In house & out house diagnostics	53	56.4	47	50.0	0.465
	Life support services	4	4.3	2	2.1	0.682
Post-hospitalization	In house drugs for 5 days	3	3.2	3	3.2	1.000
	Out house drugs for 5 days	65	69.1	46	48.9	0.008
	In house & out house drugs for 5 days	68	72.3	49	52.1	0.007

### Extent of OOPE

The median overall OOPE was $39 (Rs 2567) in the non-beneficiaries group as compared to Rs $11 (Rs 713) in the beneficiaries group (p<0.01). Median expenditure on in house and out house drugs and consumables was $ 23 (Rs 1500) in the non beneficiaries group as compared to nil in the beneficiaries group (p<0.01). Median expenditure on in house and out house diagnostics was $ 2 (Rs 150) in the non beneficiaries group as compared to $0.2 (Rs 15) in the beneficiaries group (p = 0.262). Median expenditure on in house and out house drugs post hospitalization was significantly higher in the non beneficiaries group as compared to beneficiaries group $ 3 (Rs 167) vs. $ 0.8 (Rs 50), p<0.01) (Table 4)**.**

**Table 4:** Median out of pocket expenditure (OOPE) among non-beneficiaries and beneficiaries, Solan, Himachal Pradesh, India, 2013.

	Non beneficiaries (N = 94)		Beneficiaries (N = 94)		
	Median (IQR) in USD	Range (USD)	Median (IQR) in USD	Range (USD)	p value
**Overall**					
OOPE (including all domains)	39 (4–114)	0–998	11 (4–54)	0–394	0.002
**Pre-hospitalization**					
**(1 day)**					
Drugs, consumables, diagnostics	0	0–77	0	0–23	0.795
**During hospitalization**					
In house drugs/consumables	0	0–691	0	0	0.004
Out house drugs/consumables	15 (0–65)	0–614	0 (0–25)	0–338	0.001
In house &	23 (0–94)	0–691	0 (0–25)	0–338	0
Out house drugs/consumables					
In house diagnostic	0 (0–4)	0–384	0	0–43	0
Out house diagnostics	0 (0–3)	0–384	0 (0–7)	0–323	0.217
In house & out house diagnostics	2 (0–12)	0–768	0.2 (0–8)	0–323	0.262
Life support services	0	0–31	0	0–37	0.423
**Post hospitalization**					
In house drugs for 5 days	0	0–13	0	0–8	0.996
Out house drugs for 5 days	3 (0–8)	0–38	0 (0–4)	0–28	0.009
In house & out house drugs for 5 days	3 (0–8)	0–38	0.8 (0–5)	0–28	0.008
1$ = Rs. 65.11					

### Non-beneficiary as risk factor for above median OOPE

We determined whether being non-beneficiary was an independent risk factor for above median OOPE. Among the patients with OOPE above median, proportion of non-beneficiaries was 60.6% as compared to 39.4% in the other group (p <0.01). There were significant differences (p<0.05) for the covariates namely male gender, occupation as worker, lack of knowledge regarding empanelment and median number of days of hospitalization among the patients with above median OOPE as compared to the other group (Table 5). Non-beneficiary status was significantly associated [OR: 2.4 (1.3–4.3)] with OOPE above median independently and also after adjusting for various covariates (Table 6).

**Table 5:** Beneficiary/non beneficiary status and other covariates according to the outcome variable median out of pocket expenditure (OOPE), Solan, Himachal Pradesh, India, 2013.

		OOPE > 23 $		OOPE < = 23 $		P-Value
		(Rs 1485)		(Rs 1485)		
		N = 94		N = 94		
		N	%	N	%	
**Category**	Non-beneficiary	57	60.6	37	39.4	0.006
	Beneficiary	37	39.4	57	60.6	
**Covariates**						
Gender	Male	50	53.2	32	34	0.012
	Female	44	46.8	62	66	
Education	Never attended school	23	24.5	23	24.5	0.286
	Class 1–5	13	13.8	20	21.3	
	Class 6–10	40	42.6	41	43.6	
	Higher Secondary & Above	18	19.1	10	10.6	
Occupation	Working	27	28.7	25	26.6	0
	Home maker	29	30.9	55	58.5	
	Not working	38	40.4	14	14.9	
Type of treatment	Medical	56	59.6	44	46.8	0.108
	Surgery / Obstetric	38	40.4	50	53.2	
Chronicity of Disease	Acute	59	62.8	57	60.6	0.881
	Chronic	35	37.2	37	39.4	
Type of hospital	Public sector hospital	79	84	82	87.2	0.677
	Private hospital	15	16	12	12.8	
Knowledge about empanelment of hospital	Yes	54	57.4	70	74.5	0.021
	No	40	42.6	24	25.5	
		**Median**	**IQR**	**Median**	**IQR**	
Number of days of hospitalization		5	(3–9)	4	(2–7)	0.016
		**Mean**	**SD**	**Mean**	**SD**	
Age of patient (years)		41	18.5	38	17.2	0.267

**Table 6:** Non RSBY beneficiary as risk factors for above median out of pocket expenditure (OOPE), Solan, Himachal Pradesh, India, 2013.

	OOPE > 23 $ (Rs 1485)	
	Odds ratio (OR)	95% CI
Non-beneficiary vs. beneficiary (unadjusted)	2.4	1.3–4.3
Non- beneficiary adjusted for age and number of hospitalization days as continuous variable; lack of knowledge regarding empanelment and male gender as categorical variable	3.8	1.8–8.3
Non- beneficiary adjusted for age and number of hospitalization days as continuous variable; lack of knowledge regarding empanelment, male gender, medical treatment, occupation as worker as categorical variable	4.1	1.8–9.6

### Enrollment details

All the respondents in the non-beneficiaries group were eligible for RSBY however only 67(71%) were enrolled under RSBY. The main reason for not enrolling was lack of information regarding the scheme or the organization of the enrolment camp 19(70%). Among enrolled families in the non-beneficiaries group, 24(36%) did not have their names in the card. Some of the reasons cited for this were: not present at the time of enrollment 24(33%), five members already enrolled 5(21%) and RSBY card of the family made before the marriage of the respondent 6(24%). In addition, 43 respondents had their names in the card, but could not avail the benefit as they had forgotten the card 15(35%), thought the card is not needed during pregnancy related conditions 13(30%) and due to technical reasons 15(35%).

## Discussion

RSBY achieved the objective of reducing the OOPE, improving access to health care and expanding the choice of care providers among below poverty line households in Solan, Himachal Pradesh. RSBY enabled beneficiaries to get more facilities such as drugs, consumables and diagnostics from the health facility.

The impact of HI can only be known if the enrollment of eligible beneficiaries is good as low enrollment dilutes the effect of HI. There is a wide variation in the enrollment in various Indian states with few states having enrollment as low as 21%. [6] The enrollment in Himachal Pradesh was 72%, and in district Solan 75%. This high enrollment rate provided an appropriate setting to evaluate the impact of RSBY on OOPE. The insured had half the OOPE compared to non-insured for hospitalization in our study. Different studies have reported conflicting findings regarding the effectiveness of HI in reducing OOPE and do not provide clear evidence for policy makers to support HI. Many studies in low and middle income countries like Ghana, Jordan, Mexico and India concluded that insurance reduced OOPE for health care. [2, 7–11] In contrast, a study based on national level secondary analysis of data from records of the Consumer expenditure Survey (CES), conducted by the National Sample Survey Office (NSSO) in India for pre and post insurance periods concluded the lack of financial risk protection after HI and rise in per capita expenditure on healthcare for hospitalization and increase in catastrophic headcount [12] The study showed a minimal increase in OOPE for hospitalization in post insurance period. [12] These findings need to be interpreted in context of variations in the health systems and level of programme implementation in various states The strength of our study was the design that included comparable group in a homogenous setting in same time period in terms of program implementation. Our study adds to the supportive evidence for insurance for BPL population to reduce OOPE especially in context of Himachal Pradesh, India.

A study based on CES data from NSSO for the year 1999–2000 in India had shown that the major proportion of OOPE was incurred on drugs. [13] We also had similar observations more so when the drugs or diagnostics were not available in the health facility irrespective of the insurance. This might be either due to inadequate availability of drugs, consumables and diagnostics in-house or due to lack of tie ups with outside agencies to provide a cashless experience to the patient. These gaps can be addressed by adopting better and effective procurement system for drugs and diagnostics in the public sector as have been adopted in the southern state of Tamil Nadu in India. [14] Further, standard guidelines for tie ups with outside agencies need to be formulated.

India has a rising burden of chronic diseases that require long term treatment, pushing people into poverty. [15] In our study, the respondents in the beneficiaries group were being treated for chronic conditions more often, were comparatively older and were being treated for medical conditions. This might be due to an increased health seeking behavior in the beneficiaries group, especially for chronic diseases.

RSBY reduced the OOPE but did not eliminate the OOPE. A similar finding that HI alone may not be enough to eliminate OOPE had been reported in a study conducted in states of Maharashtra and Andhra Pradesh of India. [16] An improvement in the management of health services with better efficiency and increased financing especially for drugs and diagnostics will be needed through the existing systems like NRHM to bring down OOPE for the poor. A combination of both HI and strengthened public sector may achieve the goal of universal health coverage in low resource settings.

We cannot comment on the quality of care and outcomes that might be influenced by the cost ceiling. Evidence in a well-designed study in Karnataka does point out that mortality has decreased post-insurance by a government-sponsored scheme. [17] A monitoring system, under the umbrella of RSBY to routinely capture the outcomes and quality indicators might be needed to understand the overall impact of RSBY. The limitation of our study was the information on the expenditure was based on the recall, however we restricted the study to hospitalization in the previous year and included most recent hospitalization in case of multiple hospitalizations.

RSBY decreased the extent of OOPE among the beneficiaries and enabled them to get more facilities within the hospitals; however OOPE was incurred mainly due to purchase of drugs from outside that can be further reduced by improving availability of drugs and diagnostics either by better procurement systems within the public sector or by outsourcing to the private sector. RSBY is highly relevant and need to further strengthened in context of rising burden of chronic diseases and aging population as it enhanced treatment seeking behavior among those with chronic conditions. RSBY benefits can be provided to those who fail to bring cards by alternative methods of verification such as biometrics.
